# Synergistic apoptotic effect of miR-183-5p and Polo-Like kinase 1 inhibitor NMS-P937 in breast cancer cells

**DOI:** 10.1038/s41418-021-00864-2

**Published:** 2021-09-24

**Authors:** Masahisa Kudo, Nicole Zalles, Rosario Distefano, Giovanni Nigita, Dario Veneziano, Pierluigi Gasparini, Carlo M. Croce

**Affiliations:** 1 Department of Cancer Biology and Genetics, The Ohio State University College of Medicine, Comprehensive Cancer Center, Columbus, OH USA; 2 School of Biomedical Sciences and Pharmacy, College of Health, Medicine and Wellbeing, University of Newcastle, Newcastle, NSW Australia; 3 Hunter Medical Research Institute, New Lambton Heights, NSW, Australia

**Keywords:** Cancer, Tumour biomarkers

## Abstract

MicroRNAs (miRNAs) are small noncoding RNAs that act as endogenous regulatory molecules targeting specific mRNAs for translational repression. Studies of breast cancer genomics indicate that breast cancer subtypes are distinguished and regulated by specific sets of miRNAs which affect activities such as tumor initiation, progression, and even drug response. Polo-like Kinase 1 (PLK1) is widely considered to be a proto-oncogene due to its increased expression in multiple tumor types, as well as its crucial role in regulating mitosis. Pharmacological inhibition of PLK1 can reduce tumor volume and induce tumor cell death in solid and hematologic malignancies. This prompted us to investigate how PLK1 inhibition with the target-specific inhibitor NMS-P937 would impact breast cancer cells, and how miRNAs may influence the overall response of these cells to this inhibition. We found that miR-183-5p targets PLK1 gene, effectively reducing its protein expression. Such miRNA-driven regulation of PLK1 expression sensitizes breast cancer cells to NMS-P937, resulting in synergistically increased apoptosis. We also show that the miRNA-regulated reduction of PLK1 influences the expression of apoptosis-related key proteins and possibly inducing further indirect PLK1 downmodulation through a DNMT1-p53 axis. These results suggest a potential biologically significant link between the expression of miR-183-5p and the efficacy of PLK1-specific inhibitors in breast cancer cells. Our work further elucidates how miR-183-5p regulates PLK1 gene while also enhancing NMS-P937 effect in breast cancer. Future studies assessing the role of miR-183-5p as a novel biomarker for anti-PLK1 chemotherapy agents are warranted.

## Introduction

Triple-Negative Breast Cancer (TNBC) defined by loss of expression of estrogen, progesterone, and HER-2 receptors has the highest mortality within breast cancer subtypes. TNBC is characteristically aggressive, rapidly proliferative, and has a higher propensity for metastasis when compared with other breast tumor subtypes [1–4]. Current treatment strategies are nonspecific, resulting in the poorest morbidity and mortality when compared to all the breast cancer subtypes [5–7]. One of the promising therapies in TNBC currently undergoing clinical trials is based on Polo-like Kinase 1 (PLK1) inhibitors. PLK1 is pivotal in cell cycle progression and is overexpressed in proliferative tissues, including cancer cells. Patients with tumors demonstrating higher expression of PLK1 have significantly worse outcomes [8, 9]. PLK1 has been widely considered both a novel diagnostic marker and a proto-oncogene for several tumor types, including breast cancer [10–12].

Pharmacological PLK1 inhibition in cancer has been successful in reducing tumor volume and promoting decreased viability, cell-cycle arrest, and eventually apoptosis [9, 13–17].

In this study, we utilize the PLK1 inhibitor NMS-P937 (Onvansertib) which is a highly selective and specific PLK1 inhibitor. NMS-P937 makes a series of hydrogen bonds with key residues within the ATP pocket of PLK1, inhibiting its activity and making it more prone to ubiquitination and subsequent degradation. NMS-P937 has shown substantial potency in cancer cell lines and has produced promising results in early-phase clinical trials for solid and hematologic malignancies [18, 19].

MiRNAs reduce expression levels of target genes by targeting mRNAs for cleavage and/or translational repression [20, 21]. MiRNAs have become widely considered to play crucial roles in cancer development, progression, and responses to drug treatment [22–24]. Taken together, miRNAs and gene expression can distinguish specific subtypes of breast cancer [7, 25–28] different from histological classifications. miRNAs are thus considered powerful diagnostic and prognostic biomarkers.

The goal of this study is to determine which miRNAs significantly regulate PLK1 expression via direct binding, and elucidate if this regulation affects breast cancer cells’ response to NMS-P937. These miRNAs could then serve as potential biomarkers for outcomes in patients treated with anti-PLK1 agents currently undergoing clinical testing.

## Results

### miR-183-5p directly targets the PLK1 3’ untranslated region

We utilized three miRNA-target prediction tools (miRNAMap, RNAhybrid, Targetscan) to assist in identifying PLK1-targeting miRNAs, selecting candidates predicted by at least two out of three tools (Supplementary Table 1). We chose to focus on miRNAs with dysregulated expression in breast cancer, specifically TNBC, with high-energy binding sites. We confirmed miR-18a-3p [29–31], miR-183-5p [32–34], and miR-100-5p [35–37] as candidates. RNAhybrid was used to predict high-energy binding sites in the CDS of PLK1 (Supplementary Fig. S1).

**Fig. 1:**
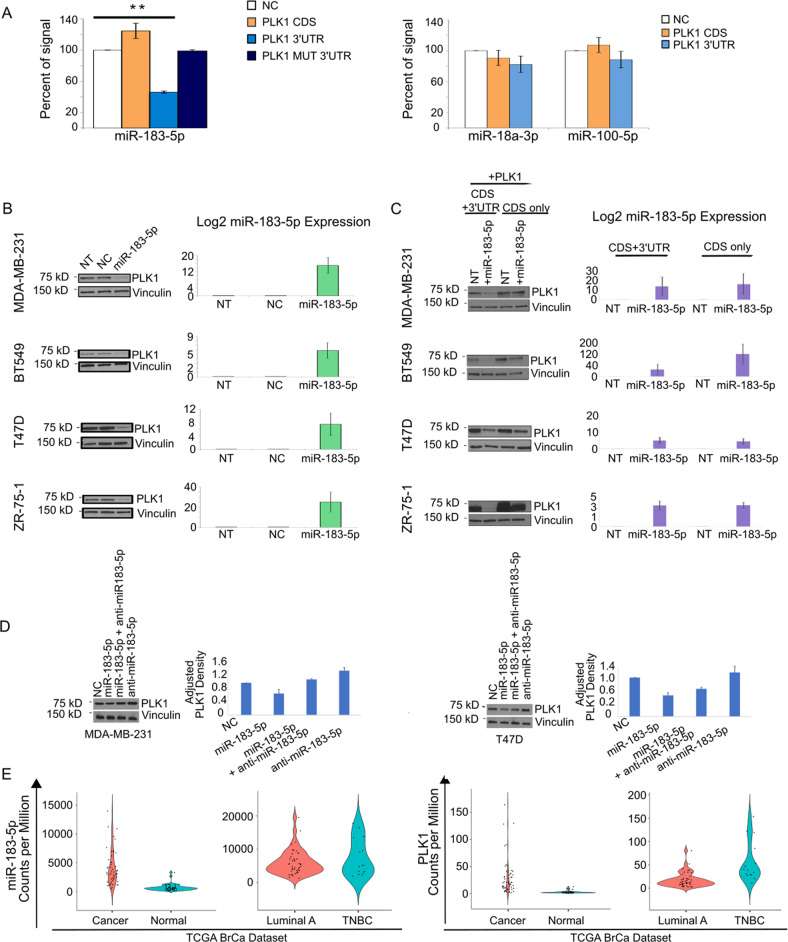
PLK1 is a direct target of miR-183-5p; PLK1 and miR-183-5p expression in TCGA Breast Cancer Dataset. **A** Left: Renilla luciferase activity analyses in HEK 293 T cells co-transfected with psiCHECK2-PLK1-CDS, psiCHECK2-PLK1-3′UTR, or psiCHECK-PLK1-3′UTR-MUT + miR-183-5p or negative control miRNA (NC) (Ambion). Results are 48 h post transfection. Right: Renilla luciferase activity analyses in HEK 293 T cells co-transfected with psiCHECK2-PLK1-CDS or psiCHECK2-PLK1-3′UTR + miR-18a-3p, miR-100-5p, or negative control miRNA (NC). Data (*n* = 3/group) are presented as mean + % SD. **B** Left: Immunoblots showing PLK1 expression following no treatment (NT), transfection with negative control (NC) or miR-183-5p. Right: RT-qPCR demonstrating expression levels of miR-183-5p following transfection (results are x10^3^). **C** Left: Immunoblots showing PLK1 expression following no treatment (NT) or miR-183-5p, following transfection with PLK1 CDS with 3′UTR (CDS + 3′UTR) or PLK1 CDS only (CDS only). Right: RT-qPCR demonstrating expression levels of miR-183-5p following transfection (results are x10^3^ for MDA-MB-231, BT549 and ZR-75-1, x10 for T47D). **D** Western blots show results of the rescue experiments (see material and methods) performed on the MDA-MB-231 (left) and T47D (right) cell lines. Bar graphs are densitometry results of PLK1-fold expression. Vinculin was used as internal control. Data are the mean of at least 3 independent experiments (*N* = 3) + SD. **p-value <0.01 compared to negative control by Student’s unpaired *t* test. **E** TCGA, IBCD analysis. Left: Violin plots represent miR-183-5p expression in Cancerous versus Normal breast tissue (*p*-value = 3.35 × 10^−21^) and in Luminal A versus TNBC (*p*-value = 0.25). Right: Violin plots depicting PLK1 expression in Cancerous versus Normal breast tissue (*p*-value = 4.22 × 10^−20^) and in Luminal A versus TNBC. (*p*-value = 1.01 × 10^−6^). *N* = 46 paired luminal A, 15 paired TNBC, 61 cancer vs normal.

The dual-luciferase reporter assay system was then used to assess the validity of these miRNAs in directly targeting PLK1. The 3′UTR and CDS of PLK1 were subcloned in the psiCHECK-2 vector (Promega, Madison, WI, USA) in two separate clones: psiCHECK-2-PLK1-3′UTR and psiCHECK-2-PLK1-CDS. MiR-183-5p was the only miRNA among the candidates that showed consistent reduction (53.6% average reduction) in luciferase activity of the psiCHECK-2-PLK1–3′UTR vector containing the miR-183-5p seed region (Fig. 1A, left panel), with no significant binding in the CDS region (Fig. 1A, left panel). No other miRNAs demonstrated significant binding in the PLK1 3′UTR or CDS (Fig. 1A right panel). To validate target binding specificity, we generated a seed region-deleted form of the 3′UTR, psiCHECK-2-PLK1-MUT-3′UTR (Supplementary Fig. S2). Co-transfection of miR-183-5p with the deleted seed form rescued the luciferase activity on ‘Wild Type’ 3′UTRs (Fig. 1A left panel).

These findings indicate that miR-183-5p specifically targets the predicted seed region in the PLK1 3′UTR.

### miR-183-5p overexpression affects PLK1 protein level

PLK1 and miR-183-5p baseline expression was determined in a panel of two TNBC (MDA-MB-231, BT549), two Luminal A (T47D, ZR-75-1) cell lines, and HMEC, normal human mammary epithelia (Clontech, Mountain View, CA USA) as reference (Supplementary Fig. S3).

After successful miR-183-5p ectopic overexpression confirmed by RT-qPCR (Fig. 1B right panel), immunoblots demonstrated markedly significant reduced PLK1 protein expression across all four cell lines (Fig. 1B left panel, Supplementary Fig. S4 top panel). miR-183-5p overexpression downregulates PLK1 mRNA levels only in BT549 and T47D cells (Supplementary Fig. S4 bottom panel).

To confirm that miR-183-5p binding was specifically responsible for the reduction of PLK1 protein, PLK1 3′UTR was inserted downstream of the PLK1 CDS expression vector (Origene, Rockville, MD, USA), generating PLK1 CDS-only and PLK1 CDS + 3′UTR expression vectors. Following transfection with these clones with or without pre-miR-183-5p (Ambion, Austin, TX, USA), immunoblotting (Fig. 1C, left panel) and densitometry-based quantification (Supplementary Fig. S5) of these cell lines demonstrated a 2.4 to 9.4-fold decrease in PLK1 protein in the cells transfected with PLK1 CDS + 3′UTR and miR-183-5p overexpression (Fig. 1C, right panel). Consistent with specific 3′UTR targeting, CDS only constructs showed a much smaller change (1.1 to 1.6-fold decrease), most likely reflecting reduction in endogenous PLK1.

To further confirm that the PLK1 reduction was strictly a result of miR-183-5p overexpression, we performed rescue experiments (see Methods for details) on two representative cell lines. Immunoblotting and densitometry of PLK1 signal (Fig. 1D left and right panels) show that PLK1 expression was reduced as a result of miR-183-5p overexpression, which is later rescued by the following inhibition of miR-183-5p, further confirming the direct effects of miR-183-5p on PLK1 protein expression.

These findings suggest that miR-183-5p regulation of PLK1 occurs exclusively via binding to its 3′UTR, and this miRNA specifically targets and reduces PLK1 protein expression in breast cancer cell lines.

### In silico analysis of miR-183-5p/PLK1 anticorrelation

PLK1 overexpression is present in various malignancies and has been considered a diagnostic as well as prognostic marker [8, 14, 38, 39]. Maire et al. demonstrated that TNBC consistently shows higher PLK1 mRNA and protein expression when compared with other breast cancer subtypes [40]. We then queried TCGA’s Invasive Breast Cancer Dataset (IBCD) for PLK1 and miR-183-5p expression, looking for possible anticorrelation in TNBC and Luminal A.

Results indicate that compared to normal breast, miR-183-5p is expressed at a higher level in breast tumors (Fig. 1E, left panel), but with no significant difference in expression between the two subtypes. Breast tumors have a higher level of PLK1 expression compared with normal breast tissue (Fig. 1E, right panel, left violin plot) and is more highly expressed in patient TNBC compared to Luminal A tumors (*p* = 1.01 × 10^−6^), this perhaps correlates with TNBC’s aggressive characteristics (Fig. 1E, right panel, right violin plot). Pearson analysis of miR-183-5p and PLK1 mRNA expression (Supplementary Fig. S6) demonstrates a positive linear correlation in unpaired normal breast tissues, and no correlation is detected in the unpaired breast tumor tissues. Spearman analysis indicates a potential positive monotonic relationship between miR-183-5p and PLK1 mRNA in normal breast tissue, but far less so in tumor tissue. We separated Luminal A and TNBC tumor samples in the initial unpaired dataset, and analyzed the paired samples. The analysis suggests a positively trending linear relationship in both Luminal A and TNBC paired “normal” tissue samples in regards to miR-183-5p and PLK1 mRNA expression. When comparing the paired breast tumor tissue samples, only TNBC samples showed a potential positive linear and/or monotonic relationship, whereas Luminal A showed little to no relationship (Supplementary Fig. S6). Regardless, the TCGA dataset analysis of PLK1 mRNA and miR-183-5p expression does not reach the expected anticorrelation as it would if the miRNA was modulating PLK1 expression at the mRNA level. We also performed Overall Survival (OS) and Relapse-Free Survival (RFS) analyses in this cohort. Stratifying for high and low expression of PLK1 and miR-183-5p, either alone or in combination, demonstrated non-statistically significant association with OS and RFS (Supplementary Table 2).

Overall, TCGA data analysis confirms the significance of PLK1 overexpression in tumor tissue compared to normal (*p* = 4.22 × 10^−20^) and its elevated expression in TNBC when compared with Luminal A cells and tissues.

### NMS-P937 synergism with miR-183-5p overexpression

We used WST8 cell viability assays (Dojindo Molecular Technologies, Rockville, MD, USA) to determine if the miR-183-5p targeting of PLK1 had any effect on the NMS-P937-induced mortality in TNBC and Luminal A cell lines.

**Fig. 2:**
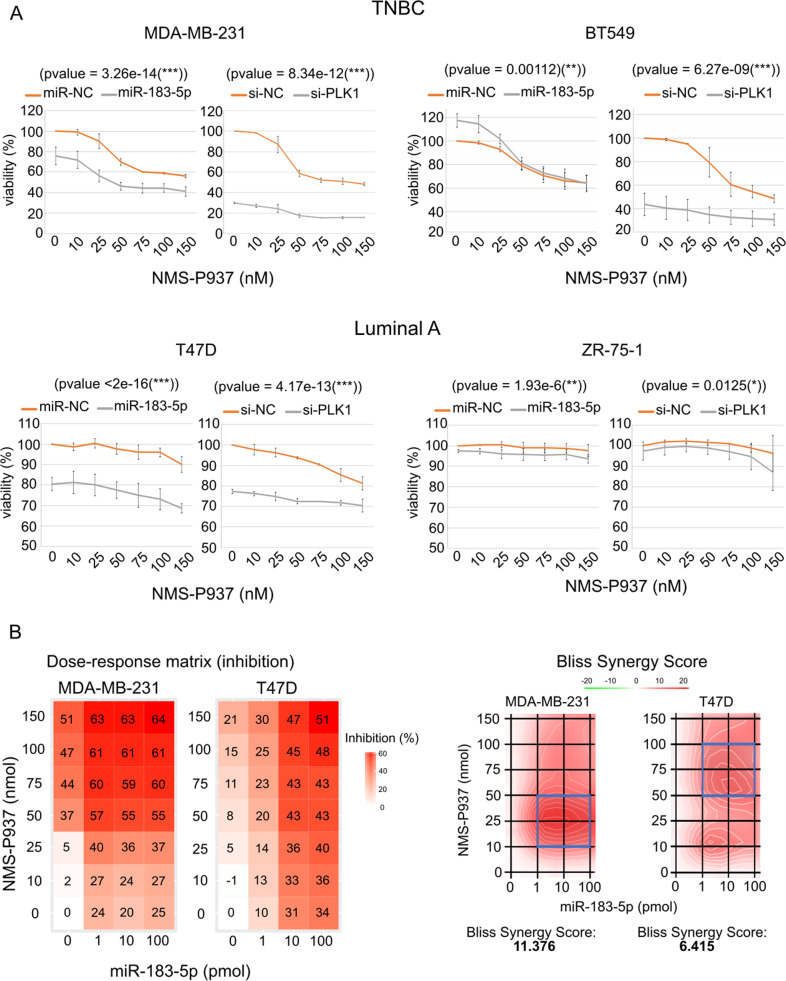
Effects of miR-183-5p on cell viability and synergistic response to combined miR-183-5p overexpression and NMS-P937 treatment. **A** WST8 assay demonstrating the effects of NMS-P937 treatment: 0, 10, 25, 50, 75, 100, and 150 nM after transfection with negative control (NC) or miR-183-5p or si-PLK1. Left hand graphs. TNBC cell lines MDA-MB-231 (top left, *p*-value = 3.26e-14) and BT549 (top right, *p*-value = 0.00112). Luminal A cell lines T47D (lower left, *p*-value < 2e-16) and ZR-75-1 (lower right *p*-value = 1.93e-6). Right hand graphs. Assay was repeated using si-PLK1. Data (*N* = 3/group) are mean + SD. **p-value <0.05, ***p-value <0.001 calculated by ANOVA. **B** Left: Matrices demonstrating MDA-MB-231 and T47D cell lines treated with different concentrations of miR-183-5p and NMS-P937, then analyzed to determine cell mortality (% inhibition). Right: Bliss score shows a synergistic effect present on cell mortality between miR-183-5p overexpression and NMS-P937 treatment.

Figure 2, panel A left side graphs show cells overexpressing miR-183-5p or miR-NC (Ambion), followed by treatment with NMS-P937; right side graphs show the same experiments using si-PLK1 (Dharmacon, Lafayette, CO, USA) to see if this highly specific PLK1 inhibitor could recapitulate the specific inhibitory action of miR-183-5p on PLK1.

In control transfected (miR-NC) TNBC cell lines MDA-MB-231 and BT549 (Fig. 2A, orange line), NMS-P937 treatment resulted in a dose-dependent reduction in cell viability, especially between 25 nM and 75 nM.

MDA-MB-231 cells overexpressing miR-183-5p and NMS-P937 treated showed significantly higher mortality compared with the cells transfected with the control only (Fig. 2A, gray line). MDA-MB-231 cells transfected with si-PLK1 recapitulate the action of miR-183-5p. Interestingly, BT549 cell line shows an opposite trend at low drug dosage, which is not shown with si-PLK1. This increased viability of BT549 cells ectopically overexpressing miR-183-5p has been previously reported [41] but this effect seems to be canceled by PLK1 inhibition via drug or si-PLK1.

Control transfected luminal A cell lines show only a minimal viability reduction after NMS-P937 treatment. T47D cell line overexpressing miR-183-5p shows a remarkable decrease in viability compared to the control, confirmed by si-PLK1. ZR-75-1 seems fairly unresponsive to treatment and to the PLK1 inhibition induced either through miR-183-5p overexpression or by si-PLK1.

Taken together these experiments show that Luminal A cell lines seem to be more resistant to NMS-P937 treatment compared to TNBC cell lines. miR-183-5p alone (NMS-P937 = 0 nM) is sufficient to decrease the cell viability in MDA-MB-231 and T47D; miR-183-5p overexpression greatly increases cell mortality following NMS-P937 treatment in MDA-MB-231 and T47D, indicating a possible additive or synergic action.

To further characterize this miRNA-drug axis, we treated MDA-MB-231 and T47D cells with increasing NMS-P937 concentration and induced miR-183-5p overexpression, analyzing the growth inhibition using the Bliss scoring method [42]. Figure 2B shows a synergistic effect with miR-183-5p overexpression and NMS-P937 treatment in the cell lines. The synergistic effect was confirmed at all concentrations of combined miR-183-5p and NMS-P937, in both MDA-MB-231 and T47D (Bliss score peak is 11.376 and 6.415, respectively).

To guarantee that the effects of miR-183-5p on cell viability are due to its direct suppression of PLK1, we assessed the ability of PLK1 re-expression to rescue the early apoptotic effects of miR-183-5p by Annexin V assay (Trevigen, Gaithersburg, MD, USA). MDA-MB-231 and T47D cells were transfected with miR-183-5p or si-PLK1, 24 h later PLK1 expression vector (Origene) was transfected to accomplish the rescue. Supplementary Fig. S7 shows that PLK1 re-expression significantly rescued the early apoptotic cell population to non-transfected levels.

Taken together these results show that miR-183-5p has a synergistic effect with NMS-P937, resulting in increased mortality of cells due to its direct PLK1 targeting.

### miR-183-5p overexpression and downstream effects of PLK1 targeting

**Fig. 3:**
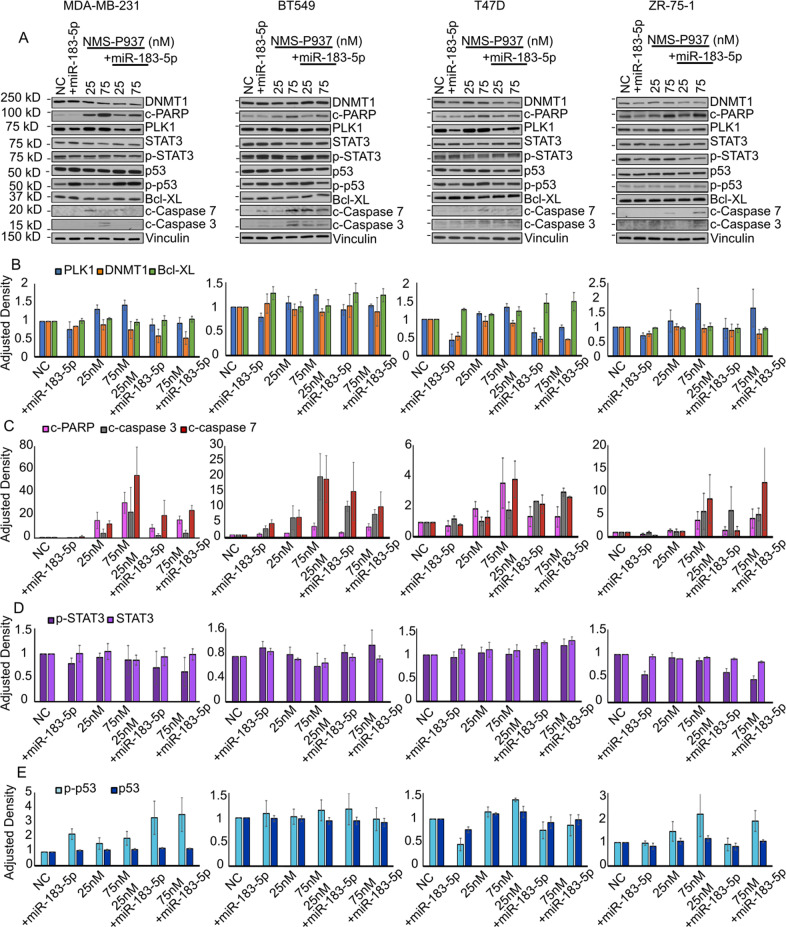
Effects of miR-183-5p overexpression on PLK1 signaling cascade and apoptosis. **A** MDA-MB-231, BT549, T47D, and ZR-75-1 cells treated with NMS-P937 for 48 h, +/- miR-183-5p transfection for 72 h. Total protein lysates were analyzed by Western blot. Blots were stained for PLK1, DNMT1, p-STAT3 (Tyr 705), p-p53 (Ser15), Bcl-XL, cleaved caspases 7 and 3, cleaved PARP and vinculin. **B**–**E** Protein band densitometry normalized to vinculin. Data (*N* = 3/group) are presented as mean + SD.

**Fig. 4:**
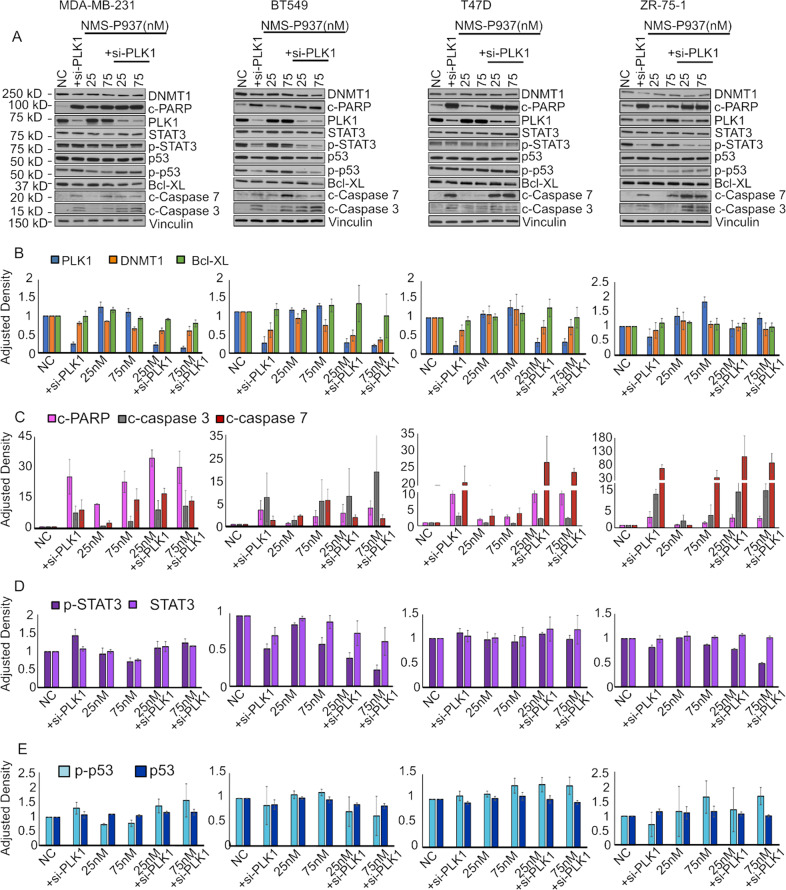
Effects of PLK1 silencing (si-PLK1) on PLK1 signaling cascade and apoptosis. **A** MDA-MB-231, BT549, T47D, and ZR-75-1 cells treated with NMS-P937 for 48 h, +/- si-PLK1 for 72 h. Total protein lysates were analyzed by Western blot. Blots were stained for PLK1, DNMT1, p-STAT3 (Tyr 705), p-p53 (Ser15), Bcl-XL, cleaved caspases 7 and 3, cleaved PARP and vinculin. **B**–**E** Protein band densitometry normalized to vinculin. Data (*N* = 3/group) are presented as mean + SD.

Given the decreased viability with miR-183-5p overexpression and treatment with NMS-P937, we decided to investigate the expression of some key proteins in PLK1 pathway together with some apoptotic markers. Figure 3 shows the changes with and without drug treatment of selected proteins after induced miR-183-5p expression. Si-PLK1 was used to determine if the changes in protein expression patterns are specifically due to miR-183-5p induced reduction of PLK1 (Fig. 4).

Immunoblot analyses show the expected reduction in PLK1 expression when cells are overexpressing miR-183-5p or si-PLK1 alone. Cells treated with NMS-P937 are consistently expressing higher or stable levels of PLK1. Although odd, this post-treatment increase has been already reported and justified as an attempted override of drug-induced inhibition through a feedback mechanism of expression of higher amounts of protein [19]. The combination miR-drug treatment does not show a synergic downregulation of PLK1 but highlights how miR-183-5p helps to counteract the feedback mechanism of PLK1 re-expression, lowering its expression compared to the drug treatment only (Fig. 3A, B blue bar). Si-PLK1 in combination with the drug reflects this behavior, greatly reducing the PLK1 expression compared to the drug only (Fig. 4A, B blue bar).

### miR-183-5p affects expression of DNMT1 and p-p53 through PLK1 targeting

DNMT1, p53, and PLK1 tightly regulate each other [43–46], thus we decided to investigate their modulation under the previous experimental conditions.

DNMT1 plays a key role in DNA methylation and is thought to play a role in the signaling mechanisms in cancer development. [47–49]. All the cell lines except BT549 show some grade of DNMT1 reduction after miR-183-5p overexpression, while the drug treatment alone seems not to change its expression. The combination miR-drug seems to have some synergic DNMT1 downregulation only in MDA-MB-231 and T47D (Fig. 3A, B orange bars). Si-PLK1 alone seems to decrease DNMT1 expression remarkably in BT549 and T47D; BT549 also shows a great synergic effect of si-PLK1 and drug combination (Fig. 4A, B orange bars).

Ingenuity Pathway Analysis (IPA) indicated DNMT1 as a predicted downstream target of PLK1. In order to make sure this effect was not a result of miR-183-5p directly binding the DNMT1 3′UTR or CDS, a luciferase assay was performed in both DNMT1 regions, but no significant binding was detected (Supplementary Fig. S8). Furthermore, in-silico analysis of isobaric Tags for Relative and Absolute Quantification (iTRAQ) relative to TCGA IBCD dataset demonstrated no significant correlation between PLK1 and DNMT1 protein expression (Supplementary Fig. S9).

PLK1 and p53 regulate each other at different levels: PLK1 prevents the phosphorylation of p53 in one or more of its activation sites, such as Ser15, to promote cell cycle progression [50, 51]; conversely, p53 is able to bind PLK1 promoter, downregulating its expression [52]. Immunoblots show that p53 maintains stable levels across the experimental conditions (Figs. 3 and 4A, E panels dark blue bar). There is an increase in p-p53 (Ser15) levels in NMS-P937-treated cells, maintained also in MDA-MB-231 and ZR-75-1 cells overexpressing miR-183-5p or si-PLK1 in combination with the drug treatment.

PLK1 and STAT3 have been described as concurrent regulators of the other’s expression and phosphorylation, either directly or as part of a complex [53–55]. Aberrant activation of STAT3 has been associated with cancer cell migration and cell cycle progression [53, 54]. MiR-183-5p alone or in combination with NMS-P937 seemed to produce no changes in STAT3 and little downregulation of p-STAT3 in MDA-MB-231 and ZR-75-1 (Fig. 3A, D light and dark purple bars, respectively) as well in the si-PLK1 transfected cells (Fig. 4A, D light and dark purple bars) only some changes in the p-STAT3 expression are detected in BT549 and ZR-75-1.

### miR-183-5p overexpression plus NMS-P937 treatment enhances the apoptotic protein cascade

Anti-apoptotic B-cell lymphoma-extra large protein (Bcl-xL) is a predicted regulatory target of PLK1 [56, 57]. Bcl-xL has been shown to influence tumor growth and proliferation by acting as an anti-apoptotic protein [58]. Bcl-xL expression in both miR-183-5p and si-PLK1 conditions seems to be mostly stable. In combination with the drug, a trend towards a small increase is seen especially in si-PLK1 transfected cells (Figs. 3 and 4A, B green bar).

The most consistent changes in expression induced by miR-183-5p and si-PLK1 with and without drug are detected in other apoptosis-related proteins, such as: c-PARP and c-Caspase-3/7 (Figs. 3A and 4A panels C pink, gray, red bars, respectively). All cell lines showed increased expression of these proteins, mostly during drug treatment alone and especially in combination with high-dose NMS-P937 and miR-183-5p or si-PLK1. Mid-late apoptosis seems to be a major drive in miR-183-5p-driven PLK1 deregulation and its synergic effects with NMS-P937.

These signal pathway results indicate that PLK1 targeting by miR-183-5p only partially influences PLK1 pathway-related protein expression, such as p53 and DNMT1, with almost no effect on STAT3, but drastically increases apoptotic proteins’ expression in TNBC and luminal A cell lines.

### miR-183-5p increases apoptosis in TNBC treated with NMS-P937

**Fig. 5:**
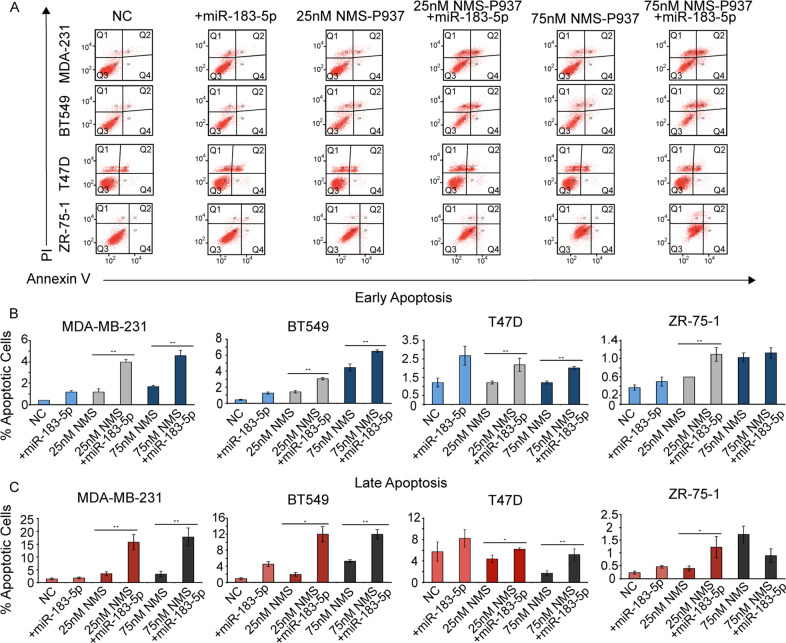
miR-183-5p + NMS-P937 Annexin V staining. **A** Representative flow cytometry figures demonstrating distribution of breast cancer cell lines treated with NMS-P937 for 48 h, ± miR-183-5p transfection for 72 h. Cells were stained with Annexin-FITC and PI. The cell populations of interest were those undergoing Late (Q2) or early (Q4) apoptosis. **B**, **C** Bar graphs representing the percentage of cells undergoing early (**B**) or late (**C**) apoptosis. Cells were either treated with negative control miRNA (NC), miR-183-5p only, NMS-P937 only, at concentrations of either 25 nM or 75 nM, or both miR-183-5p + NMS-P937. Data (*N* = 4/group) are presented as mean + SD. *p-value <0.05, **p-value <0.001 compared to negative control by Student’s unpaired *t* test.

**Fig. 6:**
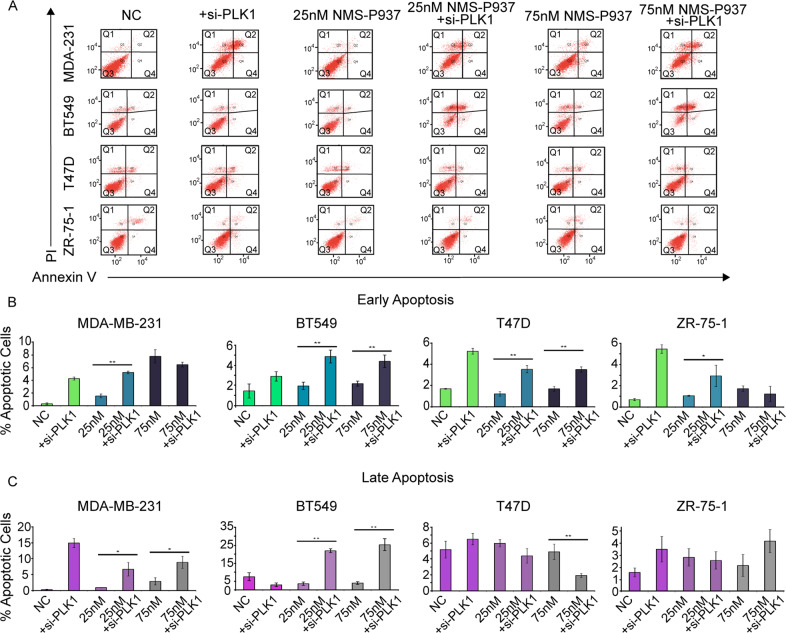
si-PLK1 + NMS-P937 Annexin V staining. **A** Representative flow cytometry images of breast cancer cell lines treated with NMS-P937 for 48 h, +/- si-PLK1 transfection for 72 h. Cells were stained with Annexin-FITC and PI. The cell populations of interest were those undergoing Late (Q2) or early (Q4) apoptosis. **B**, **C** Bar graphs representing the percentage of cells undergoing early **(B)** or late **(C)** apoptosis. Cells were either treated with negative control miRNA (NC), si-PLK1 only, NMS-P937 only (25 nM or 75 nM), or si-PLK1 + NMS-P937. Data (*N* = 4/group) are presented as mean + SD. *p-value <0.05, **p-value <0.001 compared to negative control by Student’s unpaired *t* test.

To determine the effects of the functional synergy with miR-183-5p overexpression and NMS-P937 effects on apoptosis activation, we used apoptosis-specific assay Annexin V (Trevigen), allowing us to distinguish early and late apoptosis. The early apoptosis results demonstrate that overexpression of miR-183-5p combined with NMS-P937 treatment increases apoptosis in cells; compared to NMS-P937 alone (Fig. 5A, B) very similar results were obtained with the use of si-PLK1 (Fig. 6A, B). Interestingly, in some of the untreated cell lines overexpressing miR-183-5p, the percentage of apoptotic cells was comparable to the lower concentration of NMS-P937 (25 nM) alone, confirming previous observations from the WST8 assay.

The greatest number of cells undergoing late apoptosis are miR-183-5p transfected treated with the highest NMS-P937 concentration (75 nM). MDA-MB-231 cells showed an almost 12-fold increase (1.5% to 17.8%), and BT549 cells a 13-fold increase (0.9% to 12%) of cells in late apoptosis. The miRNA + drug-treated T47D and ZR-75-1 cells showed a significant smaller increase in late apoptosis when compared with the NC (0.9-fold and 4.5-fold, respectively). Interestingly, si-PLK1 seems less effective in late apoptosis in T47D and ZR-75-1 but in general the scale at which apoptosis is occurring in the cell lines transfected with the si-PLK1 is comparable to the miRNA experiments (Fig. 6B).

Apoptosis was significantly higher in TNBC cell lines, supporting our previous observations in the viability assay. Annexin V assay highlighted an increased synergic effect with miR-183-5p overexpression and NMS-P937 treatment, exerted predominantly in TNBC cells.

Because PLK1 is a well-characterized cell cycle regulator, we wanted to confirm that cells overexpressing miR-183-5p were undergoing apoptosis rather than cytostasis. It has been reported that NMS-P937 induces G2/M block in treated cells, resulting in subsequent apoptosis [19], thus we postulated that we would see this G2/M block in breast cancer cell lines overexpressing miR-183-5p to rule out the cytostasis.

We performed cell-cycle analysis on MDA-MB-231 cells stably overexpressing miR-183-5p generated using pCDH-EF1-MCS-T2A-copGFP-PURO (System Biosciences, Palo Alto, CA, USA). The miR-183-5p stable clones treated with the highest concentration of NMS-P937 showed the highest number of cells in G2/M arrest, and a high percentage of cells with sub-G1 DNA content. These results confirm that the cooperation between miR-183-5p and NMS-P937-mediated PLK1 regulation sensitizes MDA-MB-231 cells to NMS-P937-mediated apoptosis (Supplementary Fig. S10).

## Discussion

Various studies have demonstrated the effects of PLK1 inhibition on tumor inhibition of proliferation and ultimately, apoptosis [59–61]. Preliminary and ongoing clinical trials in PLK1 inhibition as an anti-cancer treatment have shown substantial promise. The PLK1-specific inhibitor NMS-P937 has well-demonstrated anti-proliferative activity, regardless of DNA repair deficiency [18, 19].

We sought to elucidate miRNAs that directly regulate PLK1 expression, thus resulting in significant PLK1 protein expression changes. MiR-183-5p, reported to be aberrantly expressed in breast cancer, directly binds to the 3′UTR of PLK1, effectively downregulating its expression. We observed a greater response to NMS-P937 mostly in TNBC compared with Luminal A breast cancer cells. WTS8 and Bliss assays demonstrated that lowering the basal PLK1 expression in the breast cancer cell lines through miR-183-5p-mediated regulation would make them more sensitive to NMS-P937, resulting in a synergistic increase in apoptosis.

We showed that the proteins most affected by this drug-miRNA PLK1 reduction were those involved in apoptotic processes, c-PARP and c-Caspase-3/7, rather than those in the PLK1 pathway (p53, STAT3, DNMT1). Intriguingly, our results suggest a possible indirect regulation of the methyltransferase DNMT1 by miR-183-5p PLK1 targeting. This is potentially significant as DNMT1 has been shown to be necessary for cancer stem cell maintenance and tumorigenesis [48]. The detected increase in p-p53 expression following NMS-P937 treatment and miR-183-5p overexpression suggests activation of the p53 cascade, which includes the initiation of apoptosis. The observed p-p53 increase, although not striking, is intriguing since can be explained by the downregulation of its repressor DNMT1 [46]; in turn, increased p-p53 can then contribute to further PLK1 downregulation [52]. This adds a possible new indirect PLK1 downmodulation trigged by miR-183-5p overexpression.

Annexin V assay confirmed the increase in apoptosis suggested by the spike in c-PARP, c-Caspase-3/7 levels detected by Immunoblots. Annexin V highlighted the synergistic effect between miR-183-5p overexpression and NMS-P937, reflected by increased apoptosis in this combination compared to treatment conditions alone.

Interestingly, the increase in miR-183-5p and NMS-P937-mediated apoptosis was higher in TNBC compared with luminal A cells, but no successful confirmation came from TCGA’ patients data analysis.

This study shows the potential role of miR-183-5p as an onco-suppressor by inducing apoptosis through the downregulation of PLK1 and by synergically augmenting the apoptosis in breast cancer cells induced by NMS-P937. Previous research has demonstrated the tumor-suppressive characteristics of miR-183-5p in a variety of tumors [31, 62, 63]. Our results support this role and further extend it to the apoptosis pathway in breast cancer. Here we propose that miR-183-5p works synergistically with NMS-P937 not only directly affecting PLK1 expression levels. While the drug alone can induce the observed increase in PLK1 basal expression, miR-183-5p post-transcriptional regulation of PLK1 appears to bypass this mechanism, stimulating the previously described loop involving DNMT1 and p-p53. This further indirectly downregulate PLK1, thus resulting in an increase in the apoptotic cascade.

The future availability of clinical data and tissue samples from ongoing clinical trials with PLK1 inhibitors such as NMS-P937 will generate a great opportunity to assess the power of miR-183-5p as a prognostic biomarker.

## Materials and methods

### Cell culture

The human embryonic kidney cell line (HEK-293T) and human breast carcinoma cell lines were obtained from the American Type Culture Collection (Manassas, VA USA). Adherent culture HEK-293T cell line was maintained in Dulbecco’s modified Eagle’s medium (DMEM) (Life Technologies, Grand Island, NY USA) supplemented with 10% fetal bovine serum (FBS) and 1% Penicillin/Streptomycin (Life Technologies). The MDA-MB-231, BT549, T47D, and ZR-75-1 cell lines were maintained in RPMI-1640 medium (Life Technologies) supplemented with 10% FBS and 1% Penicillin/Streptomycin. All the cells were incubated in a humidified atmosphere of 5% CO_2_ in air at 37°C. All cell lines were authenticated by Short Tandem Repeat (STR) and tested for mycoplasma contamination.

### Cell line transfection and treatment

Cells were seeded in 6-well plates (1.5 × 10^5^ cells/well) a day before transfection. The transfection was performed when they were at 60–80% confluence. For overexpression experiments, cells were transfected with miR-183-5p precursor (pre-miR-183-5p) or Negative Control mimics (miR-NC) purchased from Ambion (Life Technologies). Knockdown experiments utilized SMARTpool ON-TARGET plus PLK1 siRNA (si-PLK1) (Dharmacon, Lafayette, CO USA). All transfections were performed using Lipofectamine 2000 (Invitrogen, Carlsbad, CA USA) following the manufacturer’s instructions. Analyses on recipient cells were performed 48 to 72 h after transfection. A plasmid encoding the full-length PLK1 cDNA was purchased (Origene, Rockville, MD USA; Cat.#: SC110978). Drug NMS-P937 was a kind gift of Nerviano Medical Sciences (Nerviano, Italy) and Trovagene (San Diego, CA). The drug was suspended in DMSO at a stock concentration of 10 mM, and then diluted accordingly in complete media.

### miR-183-5p rescue experiments

MDA-MB-231 and T47D were seeded in a 6-well plate, with 1.5 × 10^5^ or 3.0 × 10^5^ cells per well, respectively. On the day following seeding, the cells were transfected either with 100 pM of miR-NC (Well #1) or pre-miR-183-5p (Wells #2 and 3). The day following the transfection, 100 pM of anti miR-183-5p (Ambion, Life Technologies) was transfected into wells #3 and 4. The day following anti-miR-183-5p transfection, all cells from each well were collected for protein extraction.

TNBC cell line MDA-MB-231 and Luminal A cell line T47D were plated in a 12-well plate and transfected with 100 pM of pre-miR-183-5p (Life Technologies, Carlsbad, CA, USA) or 40 pM of si-PLK1 (Dharmacon, Lafayette, CO, USA). The next day, cells were transfected with 400 ng of the human PLK1 ORF expression vector (Origene, Rockville, MD, USA). Twenty-four hours post transfection of the PLK1 ORF vector, cells were harvested and stained with Annexin V-FITC Staining Kit (Trevigen, Gaithersburg, MD, USA) according to the manufacturer instructions and analyzed on a flow cytometer (BD LSR II flow cytometer, BD Biosciences).

### WST8 assay and bliss scoring

The WST8 cell viability assay was performed with the Cell Counting Kit-8 (Dojindo Molecular Technologies Inc. Rockville, MD USA), and the combined effect of miR-183-5p and NMS-P937 was assessed by the Bliss model analyzed with the SynergyFinder software (https://synergyfinder.fimm.fi/). TNBC cell lines and Luminal A cell lines were plated in a 12-well plate and transfected with 100 pM of pre-miR-183-5p or 40 pM of si-PLK1 for the WST8 assay, and with a concentration of pre-miR-183-5p ranging from 0 to 100 pM for the combination assay. The day following the transfection, cells were harvested and 2 × 10^3^ cells (TNBC) or 4.5 × 10^3^ cells (Luminal A) were plated into each well in a 96-well, seeding conditions were optimized to have an average 80% confluence of the cells the day the assay was performed. The next day, either regular media or drug-containing media in differing concentrations were added to the wells. After 72 h, the old media was decanted, and 5 uL of Cell Counting Kit-8 solution and 45 uL of fresh media were added per well. After 3 h of incubation at 37 °C in humidified 5% CO_2_ atmosphere, absorbance at 450 nm was measured using a SpectraMax M2 plate reader (Molecular Devices, San Jose, CA USA). Six replicate wells per time point were used to obtain measures of cell viability. Graphs represent the average of three independent experiments. *P*-values were calculated with ANOVA test.

### RNA isolation and RT-qPCR

MiRNA expression was verified by Taqman assay (Thermo Fisher Scientific, Waltham, MA USA). Total RNA from cells were isolated using TRIzol Reagent (Invitrogen) according to manufacturer’s instructions. RNA content and quality was measured using a Nanodrop-2000 (Thermo Fisher Scientific). The expression of mRNAs of interest was evaluated by the gene expression assay with the probe PLK1 Hs00983227_m1 (Thermo Fisher Scientific), and was normalized to GAPDH. Mature miRNA expression was evaluated using the single-tube TaqMan MicroRNA assay, with miRNA-183-5p probe PN4427975 (Thermo Fisher Scientific), and normalized to that of RNU44 and RNU6. All retrotranscriptase (RT) reactions, including no-template controls and RT minus controls, were run in a Veriti 96-well Thermocycler (Applied Biosystems). Each sample was tested in triplicate unless otherwise noted. Once the data from the cycling was complete, the relative quantities of miRNA and mRNA were calculated using 2^−ΔΔCT^ method after normalization.

### Immunoblotting

Cells were harvested and lysed in ice-cold RIPA lysis buffer with added protease and phosphatase inhibitor (Cell Signaling Technology, Danvers, MA USA). Equal amounts of protein were resolved and separated on an SDS/PAGE gel (BioRad TrisGlycine 4–20% gel), transferred onto PVDF membranes, and subjected to immunoblot analyses. The membranes were blocked with 5% nonfat dry milk in Tris-buffered saline, pH 7.4, containing 0.05% Tween 20, and were incubated with primary and secondary antibodies according to the manufacturer’s instructions. Blotting was performed using antibodies targeting PLK1 (Abcam, Cambridge, UK; Cat. #: 17056), DNA methyltransferase 1 (DNMT1) (BD Biosciences, San Jose, CA USA; Cat. #: 612618), Bcl-xL (Cell Signaling; Cat. #: 2764), Cleaved PARP (c-PARP) (Cell Signaling; Cat. #: 9541), p53 (DO-1) (Santa Cruz, Cat. #126), p-p53 (Ser15) (Cell Signaling; Cat. #: 9284), STAT3 (BD Biosci, Cat. #: 610189), p-STAT3 (Tyr705) (abcam; Cat. #: 76315), Cleaved Caspase-7 (c-Caspase-7) (Cell Signaling; Cat. #: 9491), Cleaved Caspase-3 (c-Caspase-3) (Cell Signaling; Cat. #: 9541), and Vinculin (Cell Signaling; Cat. # 4650). Dilutions used for Cleaved Caspase-3 and Cleaved Caspase-7 were 1:250, and for other antibodies were according to the company’s recommendations.

### Luciferase reporter assay

Plasmid generation—Luciferase constructs were all cloned between restriction sites XhoI and NotI of psiCHECK-2 vector (Promega, Madison, WI USA). PLK1 3′-untranslated region (3′-UTR) was PCR amplified from genomic DNA using two specific primers and restriction enzyme sites, XhoI (5- ccgctcgagcgggcctggagtctcctggaggaggagtacggct-3) and NotI (5-ataagaatgcggccgctaaactatttcacatctgtttaatgtgcataaagccaaggaaagg-3). PLK1 coding region (CDS) was PCR amplified using Origene plasmid (Cat#SC110978) as template and specific primers: XhoI (5-ccgctcgagcggcccggagctccggcggcggctccaccgg-3) and NotI (5-ataagaatgcggccgctaaactatgctcagcagcttgtccaccatagtgcgggc-3). DNMT1 (variant 1, NM_001130823.3) 3′UTR was PCR amplified from genomic DNA with XhoI (5- ccgctcgagcgggccaaagcccgagagagtgcctcagctaaaataaaggaggaggaagc-3) and NotI (5- ataagaatgcggccgctaaactattggtttataggagagatttatttgaagaaatattac-3). DNMT1, CDS was amplified using Origene plasmid (Cat #SC110978) as template using: XhoI (5-ccgctcgagcggatgccggcgcgtaccgccccagcccgggtgcccacactggccgtccc-3) and NotI (5-ataagaatgcggccgctaaactatctagtccttagcagcttcctcctcctttattttagc-3) as primers. All PCR products were digested with XhoI and NotI-HF (New England Biolabs) and fragments ligated into a XhoI- and NotI-digested Reporter Luciferase plasmid, psiCHECK-2 (Promega). Following confirmation of the correctly cloned sequences and subsequent expansion in transfection-competent bacterial cells (DH5α, Thermo Fisher Scientific).

The construct containing a deletion at the putative binding site (PLK1 MUT 3’UTR) was generated with primers: (5- cacactgcagacacgcgggagccaac- 3) and (5- gttggctcccgcgtgtctgcagtgtg-3) using QuikChange site-directed mutagenesis kit (Agilent Technologies, Santa Clara, CA USA).

Luciferase assay—1.5 × 10^5^ HEK-293T cells were seeded in 12-well plates overnight and then transfected with the luciferase reporter constructs along with a pre-miR-18a-3p, miR-183-5p, miR-100-5p, or miR-NC (Ambion). Cells were harvested 24 h post transfection and processed for Dual Luciferase Assay (Promega) according to manufacturer instructions.

### Stable clone generation

MiR-183-5p was amplified from genomic DNA by PCR and cloned into the Human pre-miRNA GFP Expression Lentivector pCDH-EF1-MCS-T2A-copGFP-PURO (System Biosciences, Palo Alto, CA USA) between restriction sites Xba1 and BamHI. miR-83-5p was amplified using primers correlating to these sites: Xba1 (5- gctctagagctgacccactccctccccagcctgga-3) and BamHI (5-cgggatcccggccttgaggaggagcaggctggggccctgacacaag-3). All PCR products were digested with Xba1 and BamHI (New England Biolabs) and ligated into the Xba1 and BamHI-digested expression lentivector. Following confirmation by PCR and Sanger sequencing, the recombinant miR-183-5p plasmid was packaged into mature lentivirus using pPACK Packaging System (System Biosciences) to infect 293TN cells. The supernatant of the infected cells was harvested to infect MDA-MB-231 cells. Fluorescence-activated cell sorting (FACS) was used to sort out the GFP-positive cells with the strongest miR-183-5p overexpression to establish a stably infected cell line (MDA-MB-231 + 183-5p). miR-183-5p overexpression was detected using Real-Time quantitative PCR (RT-qPCR).

### Cell cycle analysis by flow cytometry

MDA-MB-231 and MDA-MB-231 + 183-5p stable clones (2 × 10^5^ cells/well) were grown in 6-well plates for 18 h. Afterwards, the media was replaced with either plain media, 25 nM NMS-P937 or 75 nM NMS-P937 and left to incubate for another 24 h. Cells were harvested by trypsinization, fixed in ice-cold 70% ethanol, and stored at −20 °C until further analysis. For flow cytometry analysis, cells were then washed twice with ice-cold PBS, centrifuged, and resuspended in PBS containing 50 mg/ml RNaseA, 0.1% Triton X-100, and 50 mg/ml propidium iodide. Samples were then incubated at room temperature for 1 h in the dark and analyzed using a FACSCalibur flow cytometer (Becton Dickinson, Mountain View, CA). Data were analyzed using FlowJo software.

### Apoptosis analyses

Cells were detached with trypsin and washed/suspended in PBS after 48 h NMS-P937 (25 or 75 nM) treatment. The nontreated/negative tumor cells were isolated after 48 h cocultivation and suspended in PBS. The obtained cells were stained with Annexin-V-FITC Staining kit (Trevigen, Gaithersburg, MD USA) and analyzed on a flow cytometer (BD LSR II flow cytometer, BD Biosciences). Cells considered to be in early apoptosis were Annexin (FITC) positive and PI negative (Q4). Late apoptosis was defined as cells that were both Annexin V and PI positive (Q2). The percentage of cells in each category was determined by comparing the counts in each quadrant compared to the overall cell count via the FACS software. BD FACSDiva and Flowjo software were used for data analysis and figures generation.

### Biostatistical analysis of TCGA invasive breast cancer dataset

“The Cancer Genome Atlas” (TCGA) Level 3 data obtained from the TCGA Breast Cancer Project (https://portal.gdc.cancer.gov/projects/TCGA-BRCA) was employed to carry out paired differential expression and correlation analyses.

Specifically, a total of 61 paired samples (15 TNBC and 46 Luminal A) were selected for differential expression analyses.

Minimum expression filtering was applied on Mapped raw read counts (L3) of both miRNA and mRNA molecules prior to normalization, with molecules retained if their average raw read count is greater than or equal to 10 across all samples, and 33% of samples display each at least a single raw read. Filtered molecules were normalized by applying the TMM method (trimmed mean of M-values) [64]. Statistical significance of differential expression was then assessed via application of the software package EdgeR (version 3.20.9) [65].

Correlation analyses were performed on samples for which both mRNA and miRNA L3 data, as well as paired normal tissue data for both, respectively, was available (13 TNBC and 40 Luminal A samples). miRNA and mRNA data were considered as RPM (Reads Per Million) and FPKM (Fragments Per Kilobase of transcript per Million), respectively, as provided by TCGA (L3). Pearson/Spearman correlation coefficient between miRNA and mRNA data and their statistical significance was calculated by applying the software package ggpubr (version 0.2) (https://CRAN.R-project.org/package = ggpubr).

The DNMT1 and PLK1 protein expression was retrieved from the Clinical Proteomics Tumor Analysis Consortium (CPTAC) [66] database. To perform correlation for the two beforementioned genes, the 105 TCGA breast cancer samples [67] available in CPTAC were employed. The database provides protein expression acquired via the Isobaric Tags for Relative and Absolute Quantitation (iTRAQ) protein quantification methods.

### Statistical analysis

Data are expressed as the mean + standard deviation (SD) of measures used. Statistical significance was assessed by comparing mean (± SD) values with unpaired Student’s *t* test for independent groups. *P* ≤ 0.05 was considered statistically significant. GraphPad Prism software was used for statistical analysis and graph generation when indicated in the figure legends.

Supplementary information is available at Cell Death and Differentiation website.

## Supplementary information

Supplementary Table 1

Supplementary Table 2

Supplementary Figures S1-S2-S3

Supplementary Figures S4-S5

Supplementary Figures S6

Supplementary Figure S7

Supplementary Figures S8-S9

Supplementary Figure S10

Supplementary Figures and Tables legends

Raw Data TCGA Datasets

## Data Availability

All data generated and analyzed during this study are included in this published article as supplementary or from the corresponding authors on reasonable request.
