# Supplementary figures and images for: Synergistic apoptotic effect of miR-183-5p and Polo-Like kinase 1 inhibitor NMS-P937 in breast cancer cells
Source: Cell Death Differ. 2021 Sep 24;29(2):407–19. doi: 10.1038/s41418-021-00864-2 (PMC8816952; doi:10.1038/s41418-021-00864-2)

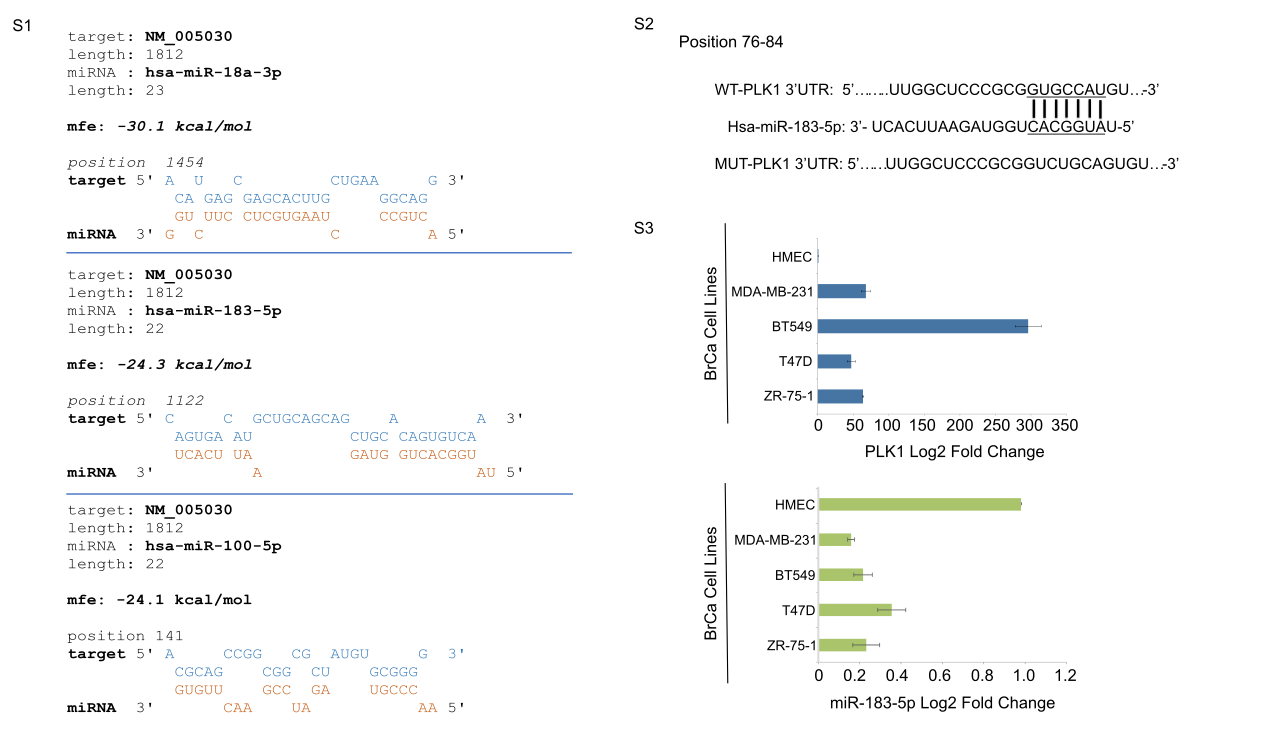

Supplement: Supplementary file 3 — Supplementary Figures S1-S2-S3 [file 41418_2021_864_MOESM3_ESM.tif]

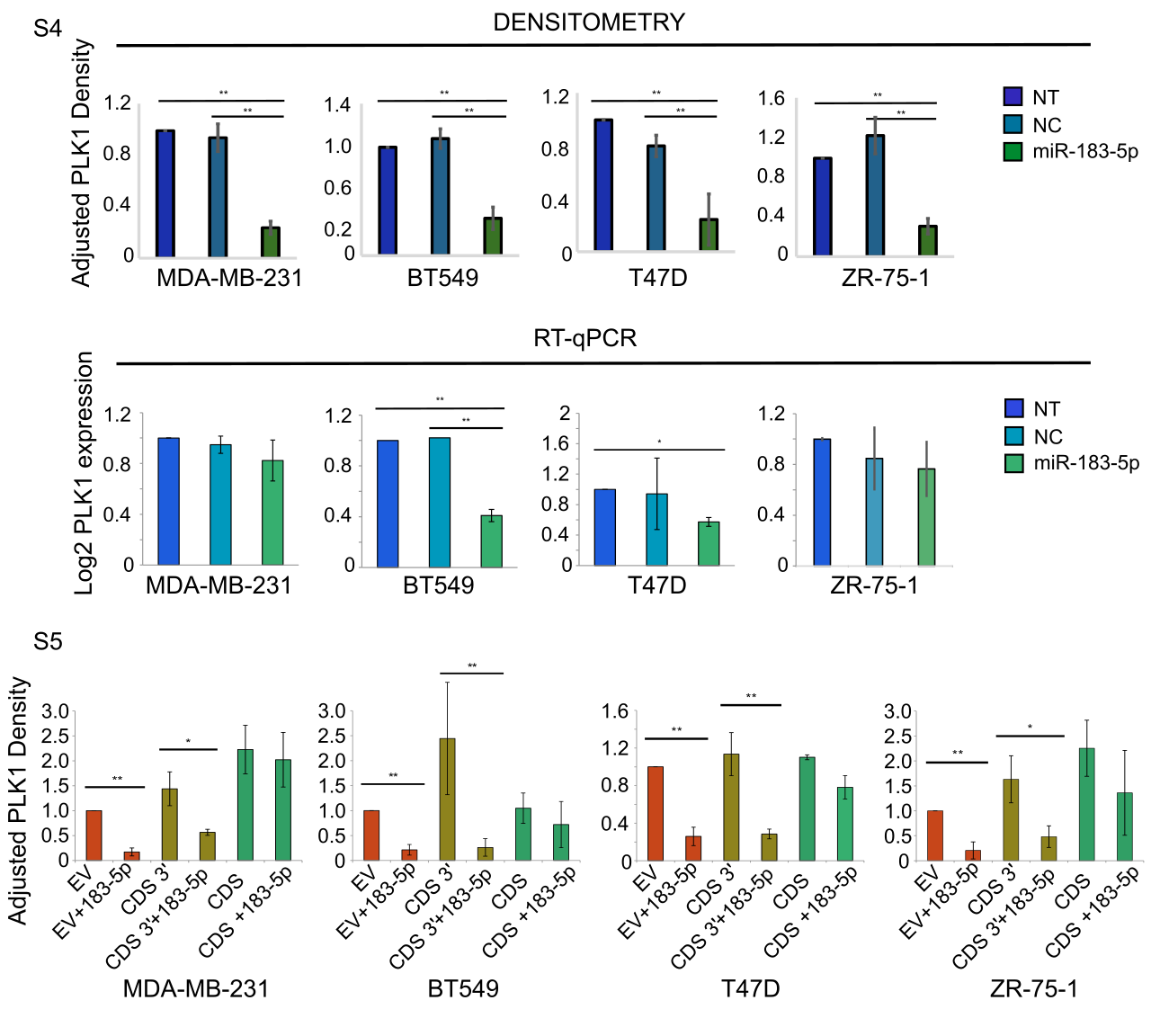

Supplement: Supplementary file 4 — Supplementary Figures S4-S5 [file 41418_2021_864_MOESM4_ESM.tif]

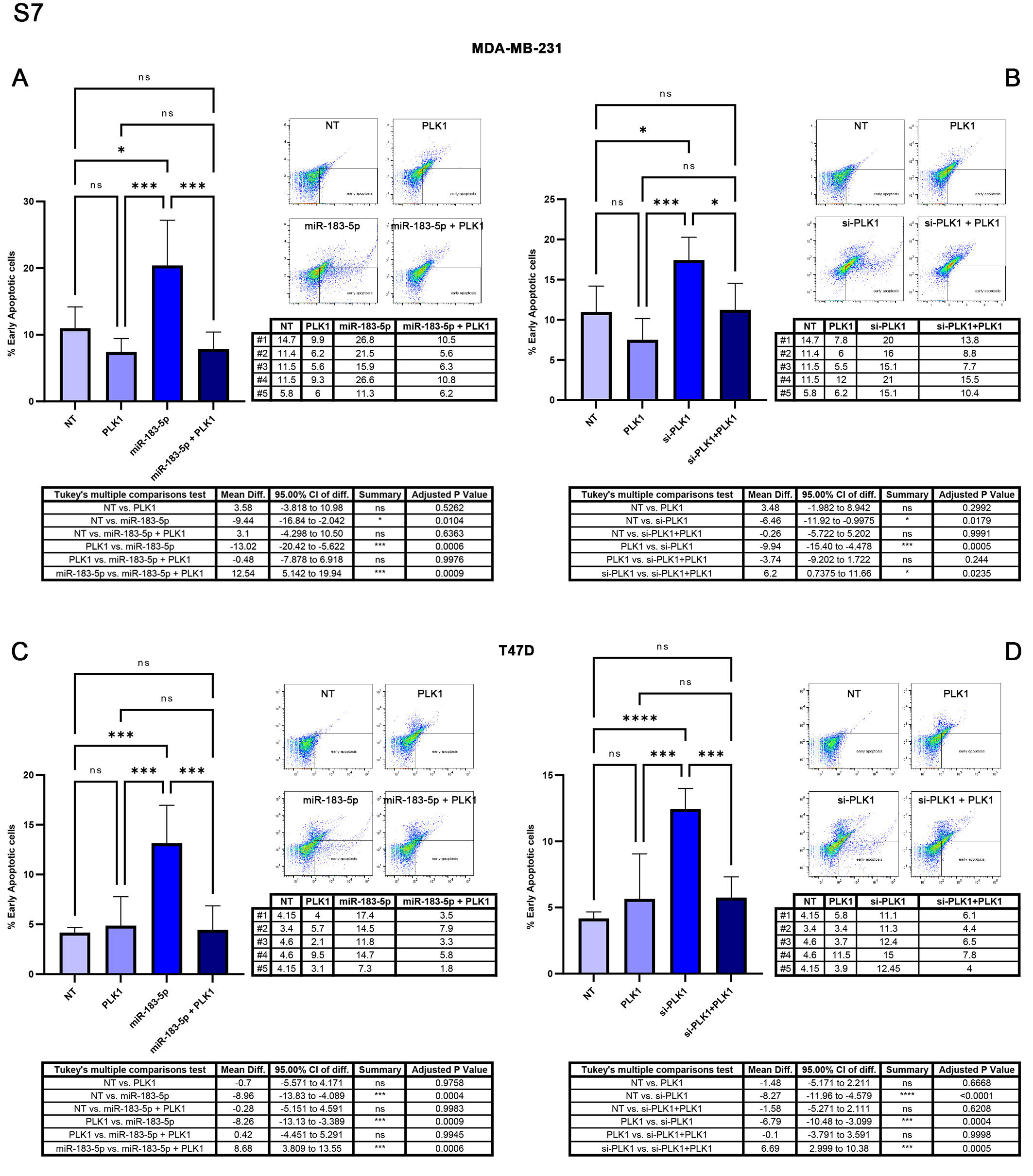

Supplement: Supplementary file 6 — Supplementary Figure S7 [file 41418_2021_864_MOESM6_ESM.tif]

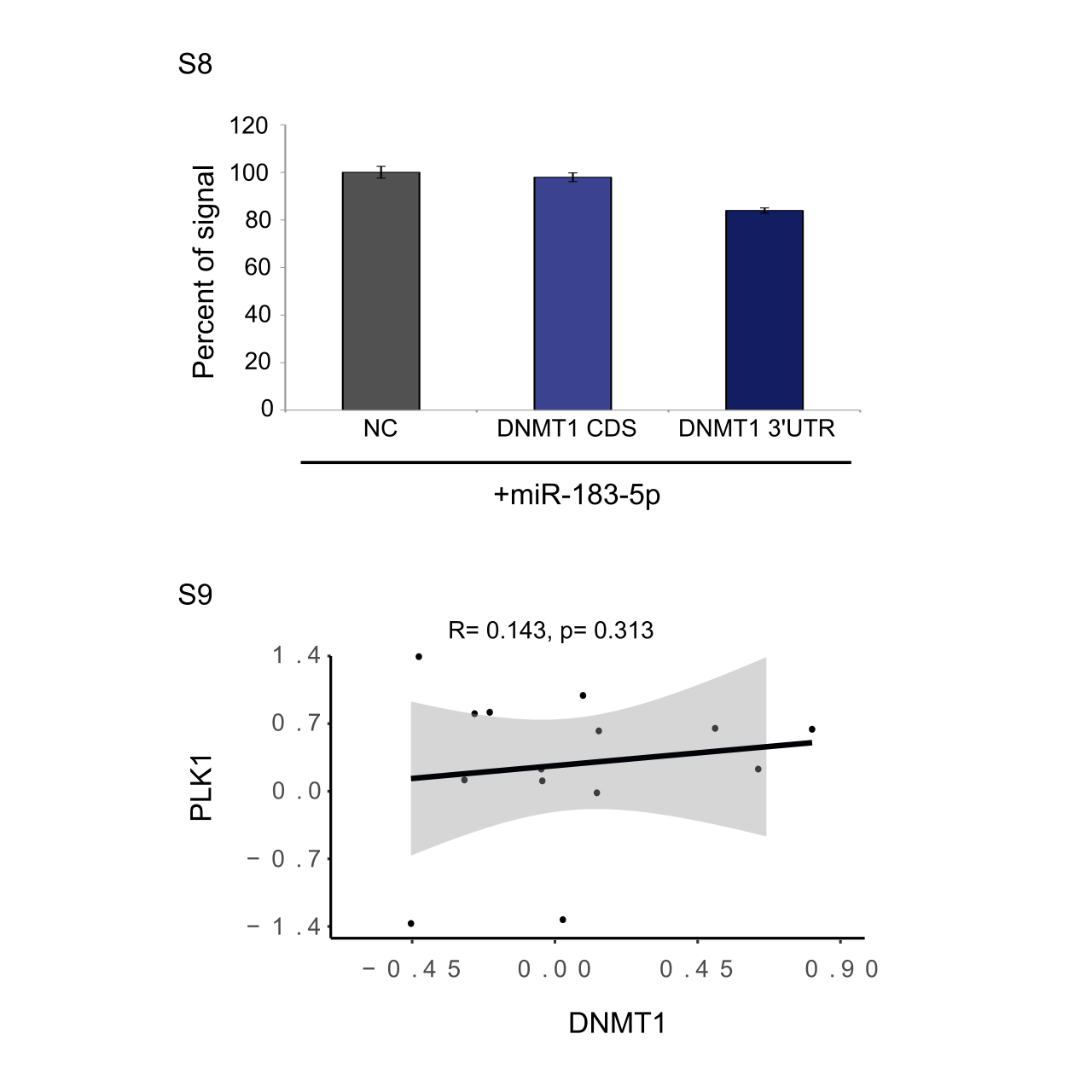

Supplement: Supplementary file 7 — Supplementary Figures S8-S9 [file 41418_2021_864_MOESM7_ESM.tif]

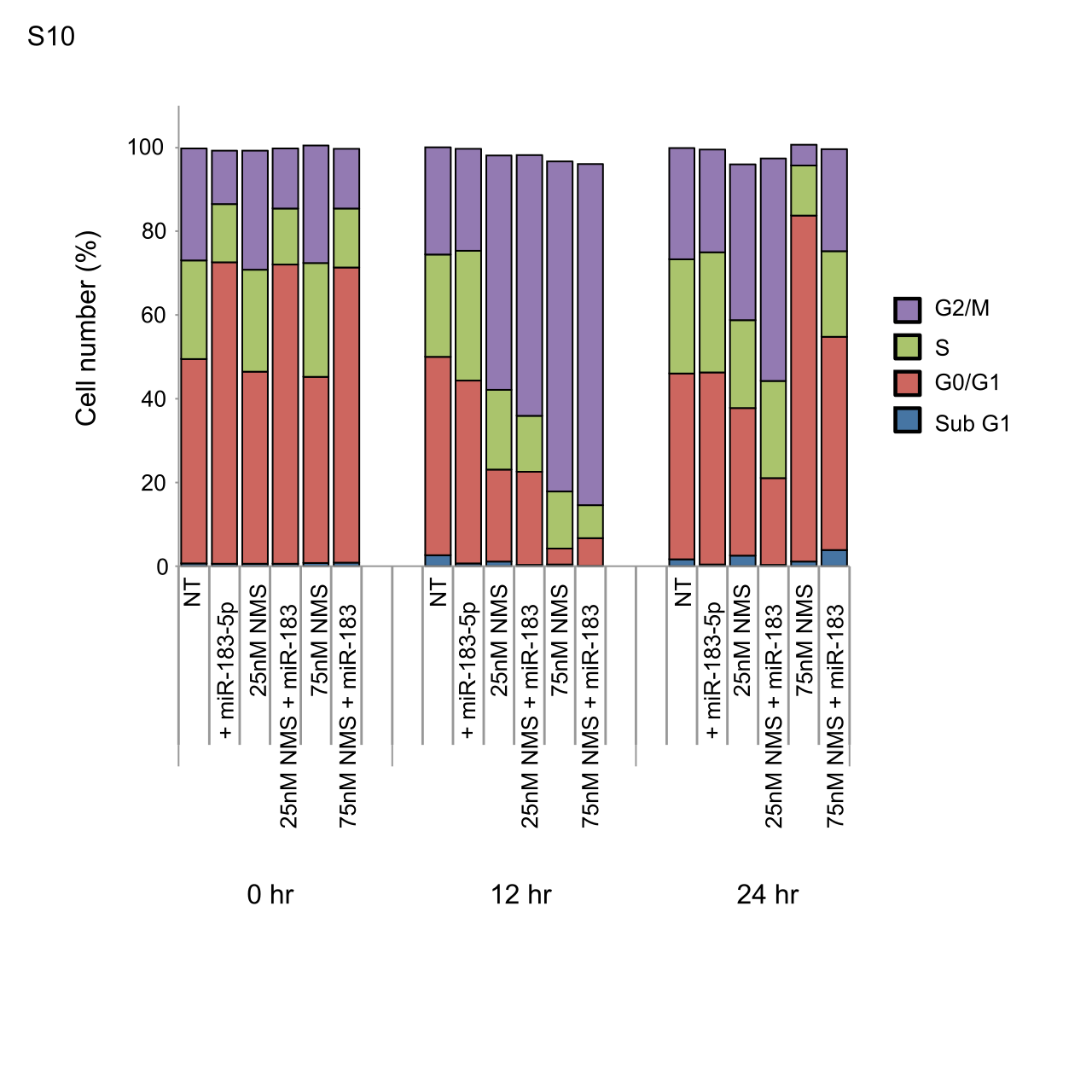

Supplement: Supplementary file 8 — Supplementary Figure S10 [file 41418_2021_864_MOESM8_ESM.tif]
