# Supplementary material for: Synergistic apoptotic effect of miR-183-5p and Polo-Like kinase 1 inhibitor NMS-P937 in breast cancer cells
Source: Cell Death Differ. 2021 Sep 24;29(2):407–19. doi: 10.1038/s41418-021-00864-2 (PMC8816952; doi:10.1038/s41418-021-00864-2)
Supplement: Supplementary file 9 — Supplementary Figures and Tables legends [file 41418_2021_864_MOESM9_ESM.docx]

**Supplementary Figure legends**

**Supplementary Figure S1. High-energy binding sites prediction in PLK1 CDS.**

RNA Hybrid prediction model of high-energy binding sites in the CDS region of PLK1 for miR-18a-3p, 183-5p, and 100-5p.

**Supplementary Figure S2. miR-183-5p seed region on PLK1 3’UTR.**

Schematic representation of the predicted human miR-183-5p binding site on the 3′UTR of PLK1 mRNA; the sequence of the deleted seed region in the psiCHECK2-PLK1-MUT-3’UTR is also shown.

**Supplementary Figure S3. PLK1 and miR-183-5p expression in BrCa cell lines.**

**Top**: RT-qPCR demonstrating baseline expression of PLK1 in human breast cancer cell lines. **Bottom**: RT-qPCR demonstrating baseline miR-183-5p expression in human breast cancer cell lines compared to normal human mammary epithelial cells (HMEC). Data (N=3/group) are presented as mean + SD. *p-value < 0.05, **p-value < 0.001 compared to negative control by Student’s unpaired t-test.

**Supplementary Figure S4. PLK1 expression following miR-183-5p transfection.**

**Top**: Densitometry of immunoblots showed in Figure 1 B normalized to vinculin. Breast cancer cell lines following 48 hours of transfection with negative control miRNA (NC) or mir-183-5p compared to non-treated (NT) cells. **Bottom**: RT-qPCR shows PLK1 mRNA levels following transfection of BrCa cell lines with miR-183-5p. Data (N=3/group) are presented as mean + SD. *p-value < 0.05, **p-value < 0.001 compared to negative control by Student’s unpaired t-test.

**Supplementary Figure S5. miR-183-5p binds only PLK1 3’UTR.**

Densitometry of Figure 1C immunoblots, showing PLK1 expression levels following transfection with Empty Vector (EV), PLK1 CDS (CDS only), PLK1 CDS with 3’UTR (CDS + 3’UTR), +/- miR-183-5p. Data (N=3/group) are presented as mean + SD. *p-value < 0.05, **p-value < 0.001 compared to negative control by Student’s unpaired t-test.

**Supplementary Figure S6. miR-183-5p and PLK1 expression in TCGA patient data.**

**Left:** Log_2_-transformed Pearson correlation data showing the relationship between PLK1 mRNA and miR-183-5p expression in the TCGA IBCD breast cancer cohort, looking for miR-183-5p and PLK1 correlation in unpaired normal breast tissue (p-value=0.025, Rho=0.31) and unpaired breast tumor tissue (p-value=0.36, Rho=0.13). Additional analysis was performed by separating the breast tumor samples into paired “normal tissue” samples of the two tumor subtypes: Luminal A (p-value=0.13, Rho=0.24) and TNBC (p-value=0.12, Rho=0.45). A potential linear relationship between miR-183-5p and PLK1 mRNA expression was evaluated for in paired breast tumor tissue samples: Luminal A (p-value=0.86, Rho=-0.029) and TNBC (p-value=0.3, Rho=0.31).

**Right:** Log_2_-transformed Spearman correlation data showing the relationship between PLK1 mRNA and miR-183-5p expression in the TCGA IBCD breast cancer cohort, looking for miR-183-5p and PLK1 correlation in unpaired normal breast tissue (p-value=0.057, Rho=0.26) and unpaired breast tumor tissue (p-value=0.5, Rho=0.094). Additional analysis was performed by separating the breast tumor samples into paired “normal tissue” samples of the two tumor subtypes: Luminal A (p-value=0.17, Rho=0.22) and TNBC (p-value=0.14, Rho= 0.43). A potential monotonic relationship between miR-183-5p and PLK1 mRNA expression was evaluated for in paired breast tumor tissue samples: Luminal A (p-value=0.83, Rho=0.035) and TNBC (p-value=0.71, Rho=0.12). Data- N= 53 unpaired samples, 40 paired luminal A samples, 13 TNBC samples.

**Supplementary Figure S7. miR-183-5p target rescue.**

In each panel clockwise: bar graphs representing the percentage of cells undergoing early apoptosis (average of 5 independent experiments). Representative flow cytometry scatter plots demonstrating distribution of early apoptotic breast cancer cell lines. Table representing the percentage of early apoptotic cells in different experimental conditions (N=5). Table below each panels summarize the ANOVA analysis (GraphPad Prism software). **A)** MDA-MB-231 were first transfected with-miR-183-5p, 24 hours later were transfected with human PLK1 ORF vector (Origene), after further 24 hours cells were collected for Annexin V assay. miR-183-5p increases early apoptosis which is rescued by the re-expression of its direct target PLK1 (statistically significant). **B)** MDA-MB-231 were transfected with si-PLK1, 24 hours later were transfected with human PLK1 ORF vector, after further 24 hours cells were collected for Annexin V assay. Si-PLK1 increases the early apoptotic cell population which is then rescued by the re-expression of PLK1 gene. **C)** T47D were first transfected with-miR-183-5p, 24 hours later were transfected with human PLK1 ORF vector, after further 24 hours cells were collected for Annexin V assay. miR-183-5p increases early apoptosis which is rescued by the re-expression of its direct target PLK1 (statistically significant) **D)** T47D were transfected with si-PLK1, 24 hours later were transfected with human PLK1 ORF vector, after further 24 hours cells were collected for Annexin V assay. Si-PLK1 increases the early apoptotic cell population which is then rescued by the re-expression of PLK1 gene. Data (N=5/group). Barr graphs are presented as mean + SD. “****” means a pvalue<0.0001; “***” means a pvalue<0.01; “*” means a pvalue<0.05; “n.s.” means that there is not significance by ANOVA test.

**Supplementary Figure S8. DNMT1 is not a direct target of miR-183-5p.**

Renilla luciferase assay data evaluating binding of miR-183-5p to the 3’UTR or CDS of DNMT1 (variant 1). HEK 293T cells were co-transfected with psiCHECK2-DNMT1-CDS or psiCHECK2-DNMT1-3’UTR + miR-183-5p or negative control miRNA (NC) (Ambion). Data (N=3/group) are mean + SD.

**Supplementary Figure S9. PLK1 and DNMT1 correlation analysis.**

isobaric Tags for Relative and Absolute Quantification (iTRAQ) of TCGA IBCD cohort was used to determine the correlation between PLK1 and DNMT1 protein expression (N= 16).

**Supplementary Figure S10. Cell cycle analysis of MDA-MB-231 stable clones.**

Graph representing percentage of stably transfected MDA-MB-231 cells collected at different timepoints following addition of NMS-P937: at immediate time of NMS-P937 treatment (0 hr), 12 hours post-treatment (12 hr), and 24 hours post-treatment (24 hr). FACSCalibur flow cytometry analysis separated cells into groups correlating with their stage in the cell cycle: G2/M, S, G0/G1 or sub G1. (N=3/group).

**Supplementary Table legends**

**Supplementary Table 1. miRNAs predicted to target PLK1 3’UTR.**

Table shows all the predicted miRNAs targeting PLK1 3’UTR, according to three miRNA-target prediction tools (miRNAMap, RNAhybrid, Targetscan).

**Supplementary Table 2. TCGA’s Invasive Breast Cancer Dataset (IBCD), Overall Survival (OS) and Relapse Free Survival (RFS) analysis.**

PLK1 and miR-183-5p, either alone or in combination, demonstrated non-statistically significant association with OS and RFS in almost all the different breast cancer subgroups. PLK1 statistically significant values are highlighted in green. See supplementary information for class comparison’s TCGA identifications.
